# Disruption of Telomere Integrity and DNA Repair Machineries by KML001 Induces T Cell Senescence, Apoptosis, and Cellular Dysfunctions

**DOI:** 10.3389/fimmu.2019.01152

**Published:** 2019-05-22

**Authors:** Dechao Cao, Juan Zhao, Lam N. Nguyan, Lam N. T. Nguyen, Sushant Khanal, Xindi Dang, Madison Schank, Bal K. Chand Thakuri, Xiao Y. Wu, Zheng D. Morrison, Mohamed El Gazzar, Yue Zou, Shunbin Ning, Ling Wang, Jonathan P. Moorman, Zhi Q. Yao

**Affiliations:** ^1^Center of Excellence for Inflammation, Infectious Disease and Immunity, James H. Quillen College of Medicine, East Tennessee State University, Johnson, TN, United States; ^2^Division of Infectious, Inflammatory and Immunologic Diseases, Department of Internal Medicine, Quillen College of Medicine, East Tennessee State University, Johnson, TN, United States; ^3^Department of Veterans Affairs, Hepatitis (HCV/HBV/HIV) Program, James H. Quillen VA Medical Center, Johnson, TN, United States

**Keywords:** aging, apoptosis, DNA damage repair, KML001, telomeres, T cells, HCV, HIV

## Abstract

T cells in chronic viral infections are featured by premature aging with accelerated telomere erosion, but the mechanisms underlying telomere attrition remain unclear. Here, we employed human CD4 T cells treated with KML001 (a telomere-targeting drug) as a model to investigate the role of telomere integrity in remodeling T cell senescence. We demonstrated that KML001 could inhibit cell proliferation, cytokine production, and promote apoptosis via disrupting telomere integrity and DNA repair machineries. Specifically, KML001-treated T cells increased dysfunctional telomere-induced foci (TIF), DNA damage marker γH2AX, and topoisomerase cleavage complex (TOPcc) accumulation, leading to telomere attrition. Mechanistically, KML001 compromised telomere integrity by inhibiting telomeric repeat binding factor 2 (TRF2), telomerase, topoisomerase I and II alpha (Top1/2a), and ataxia telangiectasia mutated (ATM) kinase activities. Importantly, these KML001-induced telomeric DNA damage and T cell senescent phenotype and machineries recapitulated our findings in patients with clinical HCV or HIV infection in that their T cells were also senescent with short telomeres and thus more vulnerable to KML001-induced apoptosis. These results shed new insights on the T cell aging network that is critical and essential in protecting chromosomal telomeres from unwanted DNA damage and securing T cell survival during cell crisis upon genomic insult.

## Introduction

T cells play a crucial role in defending the host against infections; however, the mechanisms controlling their responses to pathogenic infections remain incompletely understood. In studying the role of T cell dysfunctions in viral persistence, we and others have previously shown that chronic viral (HCV and/or HIV) infections can induce premature T cell aging and immune senescence, as evidenced by the overexpression of aging markers (such as p16^ink4a^, p21^cip1^, CD57, and KLRG1), dysregulation of aging-associated microRNAs (miR181/miR21), and particularly, accelerated erosion of telomeres, suggesting excessive cell proliferative turnover, or inadequate telomere maintenance (1–13). Telomeres are repeating hexameric DNA sequences (TTAGGG) that are found at the chromosome ends in association with a complex of shelterin proteins (14). Telomere integrity is a key feature of linear chromosomes that preserves genome stability. Conversely, telomere erosion is a hallmark of cell senescence (a quiescent, non-replicative state) that drives cell dysfunction or apoptosis (15–17).

Human T cell telomeres normally shorten at a rate of 50~100 base pairs per cell division, acting as a molecular clock to control the replicative capacity before entering cell senescence or apoptosis (17). However, telomere loss can increase up to 250 bp per cell cycle during chronic viral infection and, in compensating for this, cell cycle arrest occurs when progressive telomere loss reaches a critical point, a phenomenon known as replicative senescence (17, 18). While telomere length is maintained in most cases by an enzyme called telomerase, the telomeric shelterin complex is required to protect telomeres from the undesired DNA damage response (DDR) (16–19). The capping roles of this shelterin complex are to inhibit DDR pathways at telomeres, regulate telomerase access and functions, prevent telomeric DNA from degradation or erosion, and thus safeguard human chromosome ends (19). Among the many mammalian telomeric shelterin proteins identified in the past decade, telomeric repeat binding factor 2 (TRF2) plays a crucial role in protecting chromosome ends against instability (20, 21). TRF2 binds specifically to double-stranded telomeric sequences and remodels the telomeric DNA into a t-loop structure that is essential in protecting chromosome ends via suppressing the ataxia telangiectasia-mutated (ATM) kinase and p53-dependent DDR and non-homologous end joining (NHEJ) pathways (19, 20). In addition to its DNA capping role, TRF2 also recruits a number of proteins and enzymes required for protecting telomeric sequences against replicative DNA damages, including Apollo and topoisomerases I and IIα (Top1/2α) under topological stress (22). Notably, TRF2 expression is increased at the RNA and protein levels in a variety of human cancer cells, and its down-regulation reduces tumorigenicity (23–25). However, the role and mechanisms of TRF2, telomerase, Top1/2α, and ATM in telomeric DNA damage and repair in T lymphocyte senescence remain elusive.

Arsenic is a ubiquitous contaminant that is considered one of the top environmental health threats because of the population's potential exposure from contaminated drinking water and the high number of diseases associated with it, including cancer (26, 27). Arsenic is not considered a direct carcinogen; nevertheless, it may increase DNA damage and mutation indirectly by altering DNA repair machinery (28, 29). Recent studies have demonstrated that arsenic is also an immunotoxic agent, although the mechanisms for its cytotoxic effects remain largely unknown (30, 31). Evidence has shown that arsenic binds to telomeric DNA sequences, causing telomere attrition (32, 33). Arsenic can also induce reactive oxygen species (ROS) production, causing chromosome instability, and aberration (34, 35). On the other hand, as traditional medicine has claimed that “toxin = medicine” for centuries, arsenic compounds have been used as a treatment modality. KML001 [sodium (meta)arsenite, NaAsO2] is a telomere-targeting, orally bio-available arsenic compound with potential anti-cancer activity (32, 33).

In this study, we used KML001 as a telomere targeting agent in CD4 T cells derived from healthy subjects (HS) and chronically virus (HCV, HIV)-infected individuals to investigate the role of telomere integrity and DNA repair machineries in remodeling T cell senescence. We demonstrated that KML001 could induce telomeric DNA damage and T cell apoptosis via inhibiting TRF2, telomerase, Top1/2a, and ATM activities, similar to what we have seen in T cells during chronic viral (HCV, HIV) infection. These studies shed new light on the T cell aging network, which is important for developing novel approaches in protecting telomere integrity in T cells against chronic infectious diseases.

## Materials and Methods

### Subjects

The study protocol was approved by the institutional review board (IRB) of East Tennessee State University and James H. Quillen VA Medical Center (ETSU/VA IRB, Johnson City, TN). Written informed consent was obtained from all participants. The study subjects were composed of three populations: 26 chronically HCV-infected individuals prior to antiviral therapy, 21 latently HIV-infected individuals on antiretroviral therapy (ART) with undetectable viremia, and 64 age-matched healthy subjects (HS). Blood samples of HS, derived from Physicians Plasma Alliance (PPA), Gray, TN, were negative for HBV, HCV, and HIV infection. The characteristics of the participants in this study was summarized in Table 1.

**Table 1:** Demographic information for subjects included in this study.

**Subjects**	**Numbers**	**Age (Mean)**	**Gender (M/F)**	**Viral load and genotypes**
HCV	26	28–66 (49)	17/9	54,120 ~ 14,466,174 IU/ml, 17 GT1, 5 GT2, 4 GT3
HIV	21	28–61 (46)	15/6	All on ART with undetectable HIV-RNA
HS	64	23–65 (47)	41/23	All tested negative for HCV, HBV, and HIV

### Cell Isolation and Culture

PBMCs were isolated from whole blood by Ficoll (GE Heathcare, Piscataway, NJ) density centrifugation. CD4^+^ T cells were isolated from PBMCs using the CD4^+^ T Cell Negative Isolation Kit and a MidiMACS™ Separator (Miltenyi Biotec Inc., Auburn, CA). The isolated CD4 T cells were cultured in RPMI 1640 medium containing 10% FBS (Atlanta Biologicals, Flowery Branch, GA), 100 IU/ml penicillin and 2 mM L-glutamine (Thermo Scientific, Logan, Utah) with varying concentrations of KML001 (Sigma) or DPBS (Dulbecco's Phosphate-Buffered Saline) control for different times at 37°C and 5% CO2 atmosphere.

### Flow Cytometry

For CD4 T cell proliferation, the cells were labeled with CFSE and cultured in the presence of anti-CD3 (1 μg/ml) + CD28 (2 μg/ml, BD Bioscience) for 3 days, followed by flow cytometry analysis for measuring CFSE dilution in dividing cells as described previously (3). To quantify cell apoptosis, PBMCs or T cells were isolated, cultured in the presence or absence of KML001 and 10 μg/ml mouse anti-human CD178 (a-Fas ligand), CD40 (a-TNFα), and CD253 (a-TRAIL) or mouse IgG1 κ isotype control antibody (BD Biosciences, San Jose, CA) and collected at the indicated times, and then stained with CD4-FITC (eBioscience), Annexin V, and 7-AAD using BD Pharmingen™ PE Annexin V Apoptosis Detection Kit I (BD Biosciences). For intracellular staining, the cells were stimulated with or without 50 ng/ml PMA + 1 μg/ml Inonomycin (Sigma) and 1 μg/ml Brefeildin A (Biolegend) 4 h before harvesting, fixed and permeabilized with Foxp3 Transcription Factor Staining Buffer Set (eBioscience, San Diego, CA), and then stained with CD4-APC, IL-2-FITC (Biolegend), and IFN-γ-PE (eBioscience), TRF2-PE, or γH2AX (eBioscience). The stained cells were analyzed on AccuriTM C6 flow cytometer (BD Bioscience. Franklin Lakes, NJ) and the data were analyzed by FlowJo software (Tree Star, Inc., Ashland, OR). Isotype control antibodies (eBioscience) and fluorescence minus one (FMO) controls were used to determine the background levels of staining and adjust multicolor compensation as gating strategy.

### Telomere Length and Telomerase Activity

Telomere length of CD4^+^ T cells was measured using flow-FISH (florescence *in-situ* hybridization) protocol as described previously (4). Briefly, CD4^+^ T cells were treated with 5 μM KML001 or DPBS control for 3~5 days, and then stained with CD4-CY5 (Southern Biotech, Birmingham, AL). After fixation and permeabilization, the cells were incubated in hybridization buffer with 0.5 μM of FITC-PNA Tel C probe (CCCTAAC repeats) (PNA Bio, Thousand Oaks, CA) for 10 min at RT. Samples were heated for 10 min at 85°C, rapidly cooled on ice, and hybridized at RT in the dark overnight. Samples were washed and analyzed immediately by flow cytometry, and lymphocyte telomere length was shown as mean fluorescence intensity (MFI).

Telomeric Repeat Amplification Protocol (TRAP) assay was employed to measure telomerase activity of CD4 T cells using the TRAPEZE® RT Telomerase Detection Kit (EMD Millipore, Billerica, MA) following the manufacturer's instruction. Approximately 1 × 10^6^ CD4 T cells were purified and treated by KML001 as described above, harvested and lysed in 100 ul CHAPS buffer, incubated on ice for 30 min, and centrifuged at 12,000 g and 4°C for 20 min. About 400 ng cells lysate was applied for TRAP assay. Each sample was accompanied by two negative controls (10 min heated at 85°C or with an inhibitor). Standard curves were built on the TSR8 control template with a range of 0.04 ~ 40 amoles. About 400 ng lysate from telomerase positive cells was used as positive control. Samples were run in triplicate using the following PCR cycle conditions: 1 cycle at 30°C for 30 min and 95°C for 2 min, followed by 45 cycles at 94°C for 15 s, 59°C for 60 s and 45°C for 10 s. Data were analyzed and quantitated by CFX Manager™ Software (Bio-Rad).

### RNA Isolation and Real-Time RT-PCR

Total RNA was extracted from 1.0 × 10^6^ cells with PureLink RNA Mini Kit (Invitrogen, Carlsbad, CA), and cDNA was synthesized using the High Capacity cDNA Reverse Transcription Kit (Applied Biosystems; Foster City, CA) per the manufacturer's instruction. Quantitative PCR were run in triplicates using the following conditions: 95°C, 10 min and then 95°C, 15 s; 60°C, 60 s with 40 cycles. Gene expression was normalized to GAPDH and expressed as fold changes using the 2^−ΔΔ*ct*^ method. Primer sequences are shown in Table 2.

**Table 2:** Primer sequences for real-time RT-PCR in this study.

**Primers**	**Forward**	**Reverse**
TEF1	5′-TGCTTTCAGTGGCTCTTCTG-3′	5′-ATGGAACCCAGCAACAAGAC-3′
TPP1	5′-TCACCAGATCAGCCACATTC-3′	5′-TGGAAAGACTCTCGGAGCTG-3′
TRF2	5′-GGTACGGGGACTTCAGACAG-3′	5′-CGCGACAGACACTGCATAAC-3′
POT1	5′-TTCCACTAAAGAGCAGGCAA-3′	5′-TGAAGTTCTTTAAGCCCCCA-3′
TIN2	5′-TGCTTTCAGTGGCTCTTCTG-3′	5′-TTTACCAGCAGGTGAAGCAG-3′
RAP1	5′-TCTTCTTCAGGCAAATCTGGA-3′	5′-CCTCCTCCCAGAAGCTCAA-3′
hTERT	5′-CCAAGTTCCTGCACTGGCTGA-3′	5′-TTCCCGATGCTGCCTGACC-3′
GAPDH	5′-TGCACCACCAACTGCTTAGC-3′	5′-GGCATGGACTGTGGTCATGAG-3′
HIV RNA	5′-CAGATCCTGCATATAAGCAGCTG-3′	5′-TTTTTTTTTTTTTTTTTTTTTTTTGAAGCAC-3′

### Western Blotting

Naïve CD4 T cells were treated with 5 μM KML001 or DPBS control for 48 h. The cells were harvested and lysed on ice in RIPA buffer (Boston BioProducts Inc, Ashland, MA) in the presence of protease inhibitors (Thermo Scientific, Rockford, IL). The protein concentrations were measured by Pierce BCA protein assay kit (Thermo Scientific). The proteins were separated by SDS-PAGE, transferred to polyvinylidene difluoride membranes, pre-blocked with 5% non-fat milk, 0.1% Tween-20 in Tris buffered saline (TBS), and incubated with the following primary antibodies: PARP1, TRF1, TRF2, POT1, TIN2, RAP1, TPP1, P53, ATM, pATM, Top1, Top2a, Apollo, MRE11, RAD50, NBS1, or Sirt6. The membranes were reprobed with β-Actin antibody (Cell Signaling, Danvers, MA) as a loading control. P24 antibody was obtained from NIH/NIAID AIDS reagent program. Appropriate horseradish peroxide-conjugated secondary antibodies (Cell Signaling) were used, and the proteins were detected using Amersham ECL Prime Western Blotting Detection Reagent (GE Healthcare Bio-Sciences, Pittsburgh, PA). Protein bands were captured and quantified using the Chemi DocTM MP Imaging System (Bio-Rad System).

### TOP1cc Detection

TOP1cc was detected using the Human Topoisomerase ICE Assay Kit (Topogen, Inc., Cal No: TG1020-1, Buena Vista, CO 81211). Method of DNA purification was modified by combining ICE Assay Kit and PureLink™ Genomic DNA Mini Kit (Thermo Fisher Scientific, Catalog number: K182001, Waltham, MA 02451). Briefly, genomic DNA sample were extracted from cell pellets using the buffers of ICE assay kit and then purified by the column of PureLink™ Genomic DNA Mini Kit. The DNA samples were loaded to NC membrane by a vacuum pump, and were incubated with primary anti-TOP1cc antibody from ICE assay kit, a monoclonal antibody that specifically recognizes covalent TOP1-DNA complexes but not free Top 1 or DNA, followed by western blot procedure as described.

### Confocal Microscopy

CD4^+^ T cells were isolated and cultured as described above. The cells were fixed in 2% PFA for 20 min, permeabilized with 0.3% Triton X-100 in PBS for 10 min, blocked with 5% BSA in PBS for 1 h, and then incubated with rabbit anti-53BP1, anti-Ku70, anti-RAD51, or anti-TOP1cc antibody and mouse anti-TRF1 antibody (Cell Signaling) at 4°C overnight. The cells were washed three times with PBS with 0.1% Tween-20, stained with anti-rabbit IgG-Alexa Fluor 488 and anti-mouse IgG- Alexa Fluor 555 (Invitrogen) antibodies at room temperature for 1 h, and then washed and mounted with DAPI Fluoromount-G (SouthernBiotech, Birmingham, AL). Images were acquired with a confocal laser-scanning inverted microscope (Leica Confocal, Model TCS sp8, Germany).

### CD4 T Cells Co-culture With Huh-7.5 Cells With or Without HCV Transfection

Huh-7.5 cells were transfected with or without HCV JFH1 strain for 48 h. The HCV core protein was detected by fluorescence microscopy as described previously (36). Purified naïve CD4 T cells were co-cultured with Huh7.5 cells with or without HCV infection for 48 h. The CD4 T cells were harvested and TRF2 expression was determined by western blotting.

### HIV-1 Plasmid Transfection and Virus Infection

The pNL4-3 plasmid, which contains a full-length HIV-1 viral DNA inserted in pUC18 vector, was obtained from NIH AIDS Reagent Program. About 20 μg of the HIV-1 plasmid was used for the transfection of HEK293T cells using the polyethylenimine (PEI) method (37). The supernatants of HIV-1-transfected 293T cells were used to infect human Sup-T1 cells (obtained from an individual with Non-Hodgkin's T cell lymphoma; NIH AIDS Reagent Program) using the spinoculation method (37). Briefly, 1.5 × 10^6^ Sup-T1 cells were infected with supernatant containing 1~5 × 10^6^ HIV-1 in culture plates using centrifugation at 1,620 × g in a 37°C incubator. After 2 h of spinoculation, the supernatants were removed to get rid of the unattached viruses. Cells were harvested at the indicated times, and used for RT-PCR and western blot analyses.

### Statistical Analysis

The data were analyzed using Prism 7 software, and are summarized as mean ± SEM or median with interquartile range. Comparisons between two groups were made using independent Student's *t*-test, or paired *T* test. Multiple comparisons were made using test/least significant difference or Tukey's procedure, depending on the ANOVA F test or by a non-parametric Mann–Whitney *U*-test. *P*-values < 0.05, < 0.01, or < 0.001 were considered statistically significant or very significant, respectively.

## Results

### KML001 Inhibits Human CD4 T Cell Proliferation, Cytokine Production, and Induces Apoptosis

**Figure 1:**
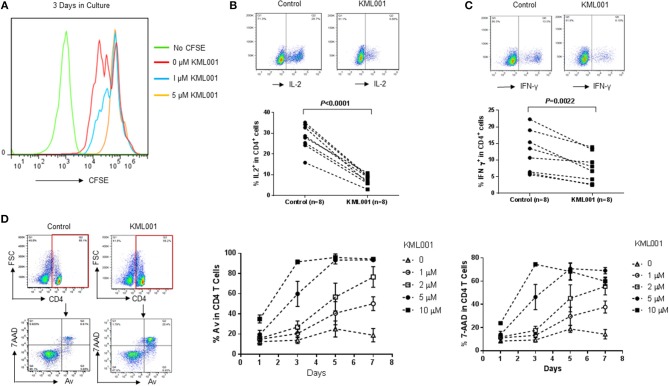
KML001 inhibits CD4 T cell proliferation, cytokine production, and induces apoptotic death. Healthy PBMCs were cultured in the presence or absence of TCR stimulation and varying concentrations of KML001 for different times, followed by measuring T cell proliferation, cytokine production, and apoptosis by flow cytometry. **(A)** KML001 inhibits CD4 T cell proliferation in a dose-dependent manner, measured by CFSE dilution in dividing cells. **(B,C)** KML001 inhibits IL-2 and IFN-γ productions in TCR-stimulated CD4 T cells. Representative dot plots and summary data from 8 subjects per group are shown. **(D)** KML001 promotes CD4 T apoptotic death, in a dose- and time-dependent manner, determined by the percentage of Av/7AAD positive cells.

Environmental exposure of human populations to arsenic from contaminated drinking water has been associated with a high incidence of detrimental diseases, likely due to its immunotoxic and genotoxic effects (25–35). As a first step to study its effects on the human immune system, we employed arsenic KML001—a telomere-targeting drug—as a tool by culturing healthy PBMCs in the presence or absence of anti-CD3/CD28 (1 μg/ml each) stimulation and varying concentrations of KML001 for different times, followed by measuring T cell proliferation, cytokine production, and apoptosis by flow cytometry. As shown in Figure 1A, TCR-stimulated CD4 T cells exhibited better cell division (measured by CFSE dilution) when cultured for 3 days in the absence of KML001 (red line), moderate cell division at low dose (1 μM) of KML001 (blue line), and no cell division in the presence of high dose (5 μM) of KML001 (orange line), indicating a dose-dependent inhibition of T cell proliferation by this telomere-targeting drug. Also, the intracellular IL-2 (Figure 1B, *P* < 0.0001) and IFN-γ (Figure 1C, *P* = 0.0022) cytokine productions in TCR-stimulated CD4 T cells were significantly inhibited by KML001 treatment for 48 h. Moreover, PBMCs exposed to KML001 showed dose- and time-dependent increases in CD4 T cell apoptotic death compared to the untreated controls (Figure 1D). These data suggest that KML001 inhibits T cell proliferation, cytokine production, and promotes cell apoptotic death.

### KML001-Induced T Cell Apoptosis Is Independent Upon the Extrinsic Death Pathways

**Figure 2:**
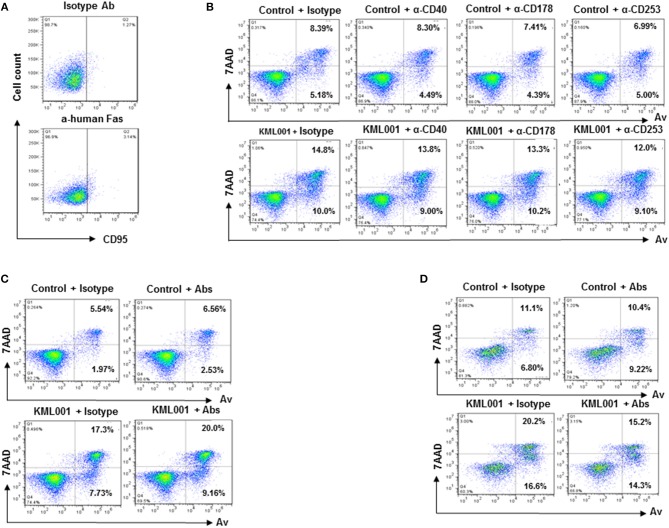
KML001-induced CD4 T cell apoptosis is independent of the extrinsic death pathways. **(A)** CD95 (Fas receptor) expression on resting naïve CD4 T cells, measured by flow cytometic analysis. **(B)** Purified CD4 T cells were cultured with or without 5 μM KML001 in the presence of blocking antibodies for Fas ligand (10 μg/ml), TNFα (10 μg/ml), and TRAIL (10 μg/ml), or IgG1 κ isotype control for 48 h, followed by Av/7AAD assay to determine cell apoptosis. **(C)** Purified CD4 T cells were cultured with or without 5 μM KML001 in the presence of all three blocking antibodies, followed by Av/7AAD assay. **(D)** PBMCs were cultured with or without 5 μM KML001 in the presence of all three blocking antibodies or isotype control, followed by Av/7AAD assay.

The mechanisms underlying KML001 induction of T cell apoptosis remain unclear. Interactions between Fas-Fas ligand, TNFα-TNF receptor, and TRAIL-TRAIL receptor have been shown to play an important role in cell apoptosis in response to exogenous or endogenous stimulations. Notably, we and others have previously shown that in chronic viral or inflammatory diseases, the problem of T cell homeostasis is primarily vested in naïve T cell loss; however, resting naïve T cells usually do not express these receptors on their cell surface to initiate programmed cell death (Figure 2A) (36, 38, 39).

To determine whether KML001-induced T cell apoptosis is dependent upon the extrinsic death pathways, we incubated purified CD4 T cells with or without 5 μM KML001 in the presence of blocking antibodies for Fas-ligand (10 μg/ml anti-human CD178), TNFα (10μg/ml anti-human CD40), and TRAIL (10μg/ml anti-human CD253), or IgG1 κ isotype control antibody (BD) for 48 h, followed by Av/7AAD assay to determine cell apoptosis. As shown in Figure 2B, compared to the controls (Control + Isotype), CD4 T cells incubated with the blocking antibody alone in the absence of KML001 (upper panel) did not exhibit any change in cell apoptosis and death. KML001 treatment (KML001 + Isotype) increased T cell apoptosis and death; however, adding blocking antibody into the culture (lower panel) did not abrogate the KML001-induced T cell apoptosis and death. Additionally, we added all three blocking antibodies simultaneously into the cultures and did not observe any antagonistic effect on KML001-induced T cell apoptosis and death (Figure 2C), although these blocking antibodies slightly diminished the KML001-induced cell apoptosis in cultured PBMCs (Figure 2D), which include NKs, B cells, and monocytes/macrophages that may have respective ligand/receptor expressions on their cell surfaces. We were able to reproduce these results using CD4 T cells or PBMCs isolated from different subjects. Taken together, these results suggest that blocking the extrinsic death pathways by disrupting the Fas-Fas ligand, TNFα-TNFγ, and TRAIL-TRAILγ interactions in cultured CD4 T cells does not affect KML001-mediated cell apoptotic death. This is in line with the reports showing that naïve CD4 T cells are resistant to exogenous apoptotic pathway-mediated cell death, but sensitive to endogenous oxidative stress, being particularly vulnerable to ROS-mediated genotoxicity (36, 38–40).

### KML001 Induces T Cell Apoptotic Death Through the Telomeric DNA Damage Response (DDR)

**Figure 3:**
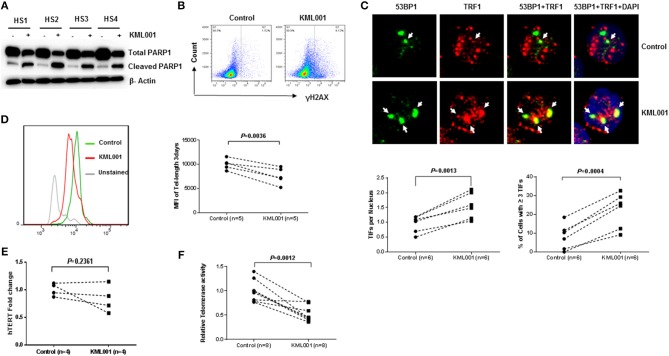
KML001 triggers telomeric DDR and causes telomere attrition in CD4 T cells. **(A)** Purified CD4 T cells from HS were treated with 5 μM KML001 or DPBS control for 48 h, followed by western blotting to measure total and cleaved PARP1 levels. **(B)** KML001 induces γH2AX expression in CD4 T cells exposed to 5 μM KML001 for 48 h, followed by flow cytometry analysis. **(C)** KML001 increases dysfunctional telomere-induced foci (TIF). Purified CD4 T cells were treated with 5 μM KML001 or DPBS control for 48 h, followed by confocal microscopy examination to assess co-localization of 53BP1 and TRF1. Approximately 100 cells were counted, and representative imaging and summary data of the TIF numbers per nucleus and the percent of cells with ≥ 3 TIFs are shown. **(D)** Purified CD4 T cells were treated with 5 μM KML001 or DPBS control for 5 days, followed by Flow-FISH analysis to determine telomere lengths. Representative overlaid histograms and summary data are shown. **(E)** Purified CD4 T cells were treated with 5 μM KML001 or DPBS control for 48 h, followed by real-time RT-PCR to measure hTERT (the catalytic unit of telomerase) mRNA levels. **(F)** Telomerase activity in CD4 T cells treated with 5 μM KML001 or DPBS control for 48 h, followed by TRAP assay. The number of subjects to be examined and the *p*-value of statistical analysis is shown in all summary data.

How KML001 triggers T cell apoptosis is unclear. Previous studies have shown that KML001 can directly bind to telomeric DNA sequences and cause cytotoxicity by inducing telomere erosion in cancer cells (32). To investigate whether KML001 triggers T cell apoptosis via telomeric DNA damage or telomere erosion, we examined DNA damage markers and telomere length in CD4 T cells exposed to it. We chose to study caspase-dependent pathways as they are the central components of the apoptotic response. Caspases are usually produced in cells as catalytically inactive zymogenes and must undergo proteolytic activation before entering the apoptotic cascade (41). For example, caspase-3 is frequently activated by death proteases to catalyze the specific cleavage of cellular proteins including Poly ADP-Ribose Polymerase 1 (PARP1), an enzyme that catalyzes the transfer of ADP-ribose onto target proteins that plays an important role in maintaining DNA damage repair and chromosomal stability (41). Therefore, to determine whether KML001-induced T cell apoptosis is mediated by triggering telomeric DDR, we measured the total and cleaved form of PARP1 in KML001-treated CD4 T cells by western blotting and found that the total level of PARP1 was significantly decreased, whereas its cleaved form was significantly increased, in CD4 T cells treated with 5 μM KML001 for 48 h (Figure 3A). This data clearly reveals a DNA damage-triggered, capspase-3-dependent T cell apoptosis induced by the KML001 treatment.

Following genomic insults, histone variant H2AX is phosphorylated (S139) at the site of DNA double strand breaks (DSB) to form γH2AX that subsequently recruits other DNA damage and repair proteins to trigger DDR. To further assess telomeric DDR as a possible cause of impaired T cell survival, CD4 T cells were purified from HS, treated with 5 μM KML001 for 48 h, followed by flow cytometric analysis of γH2AX, a marker for DNA damage. As shown in Figure 3B, γH2AX expression was remarkably increased in the CD4 T cells exposed to KML001.

In conjunction with H2AX phosphorylation, 53BP1 is recruited to the DNA damage site (including telomeric break vicinity) and acts as a docking site for other adaptor proteins to form microscopically visible nuclear focus (DNA damage foci). Thus, identifying dysfunctional telomere-induced foci (TIF) is typically deemed as a hallmark of telomeric DDR (42, 43). To confirm telomeric DNA damage in CD4 T cells exposed to KML001, we compared numbers of TIFs per nucleus and the percent of cells with > 3 TIFs by examining co-localization of 53BP1 with telomere-associated shelterin protein TRF1 by confocal microscopy. As shown in Figure 3C, the representative imaging and summary data of confocal microscopy, the numbers of 53BP1/TRF1 TIF per nucleus and the percentage of T cells with > 3 TIFs were significantly higher in CD4 T cells treated with KML001 for 48 h (*P* = 0.0013, *P* = 0.0004, respectively). These data indicate that KML001-treated CD4 T cells exhibit DNA damage that extends to the chromosome ends—telomeres.

### KML001 Induces Telomere Erosion by Inhibiting Telomerase Activity

Telomere erosion is a hallmark of cell senescence that leads to cell dysfunction or apoptosis (15–17). To demonstrate whether KML001-treated human CD4 T cells have telomere erosions, we measured telomere length by Flow-FISH. As shown in Figure 3D, purified human CD4 T cells treated with 5 μM KML001 for 3 days exhibited a significantly shortened telomere length compared to the DPBS-treated control (*P* = 0.0036). These results recapitulate the findings by us in patients with chronic viral (HCV, HIV) infection in that their T cells are senescent, characterized by the significant telomere loss (4, 38).

Because of the inability of conventional DNA polymerase to replicate the 3′-end telomeric overhang, telomere sequences are replenished by telomerase, which is composed of telomerase RNA (TR) and human telomerase reverse transcriptase (hTERT)—the catalytic unit of telomerase (44). Since the primary function of telomerase is to prolong telomeric DNA sequences, we hypothesized that T cell telomere shortening is likely a result of telomerase inhibition by KML001. To test this possibility, we measured hTERT transcript copies in purified CD4 T cells treated with 5 μM KML001 or DPBS for 48 h using real-time RT-PCR. Unexpectedly, no significant differences (*P* = 0.2361) were found in hTERT expression levels in T cells exposed to KML001 or DPBS control (Figure 3E). We then measured the telomerase activity by TRAP assay in CD4 T cells under the same treatment conditions. As shown in Figure 3F, KML001-treated T cells exhibited significantly decreased telomerase activity, suggesting that KML001 causes telomere loss, not by inhibiting the hTERT expression, but via inhibiting its enzymatic activity. Together, these results indicate that KML001-induced telomeric DNA damage and telomere erosion may cause cell apoptosis and T cell loss, emphasizing the role of telomere integrity in securing T cell survival.

### KML001 Disrupts Telomere Integrity by Inhibiting Shelterin Protein TRF2 Expression

**Figure 4:**
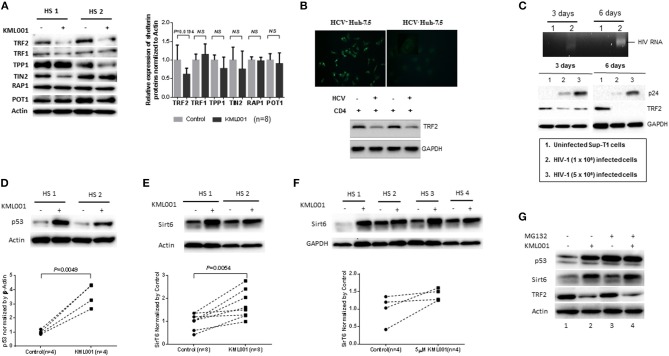
KML001 impairs telomere integrity by inhibiting TRF2 protein expression. **(A)** Purified CD4 T cells were treated with 5 μM KML001 or DPBS control for 48 h, followed by western blotting to measure TRF2, TRF1, TIN2, POT1, TPP1, PAP1 protein expressions. Representative imaging and summary data of the western blot are shown. NS, not significant. **(B)** Upper panel, HCV core protein was detected by fluorescence microscopy in Huh-7.5 hepatocytes transfected with HCV-JFH1 strain, but not in the mock transfected cells. Lower panel, TRF2 was inhibited in CD4 T cells co-cultured with HCV^+^ Huh-7.5 cells compared to those incubated with HCV^−^ hepatocytes. GAPDH served as a loading control. **(C)** Upper panel, RT-PCR detection of HIV-RNA in SupT1 cells at day 3 and day 6 of HIV-1 infection. Lower panel, western blotting of HIV-1 p24 and TRF2 expressions in SupT1 cells without (lane 1), or with low (lane 2) and high (lane 3) dose of HIV-1 infection. GAPDH served as loading control. **(D,E)** Western blot analysis of p53 and Sirt6 expressions in CD4 T cells exposed to KML001 (5 μM) or DPBS control for 48 h. Summary data were normalized to the DPBS control. **(F)** Western blot analysis of Sirt6 expression in TCR-activated CD4 T cells by anti-CD3/CD28 stimulation (1 μg/ml) and exposed to KML001 (5 μM) or DPBS treatment for 48 h. **(G)** Western blot analysis of p53, Sirt6, and TRF2 expressions in CD4 T cells with or without KML001 treatment in the presence or absence of proteasomal inhibitor (MG-132).

Since telomeres are nucleoprotein complexes with a primary function of capping the chromosome ends to prevent unwanted DNA damage, we hypothesized that disrupting telomere shelterin may uncap chromosome ends, leading to telomere deprotection, and telomere erosion. To test this possibility, we treated human CD4 T cells with 5 μM KML001 or DPBS for 48 h, followed by measuring the levels of shelterin proteins by western blotting. As shown in Figure 4A, among the known human shelterins (includes six polypeptides: TRF1, TRF2, RAP1, TIN2, TPP1, and POT1), only TRF2 expression was significantly inhibited (*P* = 0.0194) in CD4 T cells following KML001 treatment. TPP1 (*P* = 0.1296) and TIN2 (*P* = 0.2230)—the two proteins that can form a sub-complex with TRF2—were also slightly inhibited, whereas TRF1 (*P* = 0.2550), RAP1 (*P* = 0.5423), and POT1 (*P* = 0.2994) protein levels remained unchanged by the treatment. These results are similar to our findings in CD4 T cells derived from chronic HCV or HIV infection (38).

TRF2 appears to be central to telomere integrity in both the setting of KML001 treatment and during viral infections such as HCV and HIV. Since chronically HCV-infected patients often have other co-morbidities that may interfere with TRF2 levels and cause immune dysregulation, we examined the specific role of HCV in inhibiting TRF2 expression. To this end, we incubated naïve CD4 T cells with Huh7.5 cells with or without HCV infection, followed by measuring TRF2 protein expression. As shown in Figure 4B, hepatocytes transfected with HCV-RNA for 48 h showed positive HCV core protein by immunofluorescence staining, whereas cells with mock transfection exhibited negative staining. RT-PCR showed HCV RNA in the supernatant of the HCV-transfected cells, but not in media of mock-transfected cells (data not shown). Importantly, similar to the findings in CD4 T cells derived from HCV patients or those treated with KML001, TRF2 protein was suppressed in CD4 T cells co-cultured with HCV^+^ Huh-7.5 cells compared to those incubated with HCV^−^ Huh-7.5 hepatocytes, suggesting that HCV, like KML001, can inhibit TRF2 expression.

Also, we found that TRF2 is inhibited, at the posttranscriptional level, in CD4 T cells derived from HIV-infected individuals, we next examined the specific role of HIV in inhibiting TRF2 expression in the absence of other co-founding factors. To this end, we established an *in vitro* HIV-1 T cell culture system by employing a pNL4-3 plasmid (from NIH AIDS Reagent Program) that contains a full-length HIV-1 viral genome (45). Culture supernatants containing 1~5 × 10^6^ HIV-1 virions from HEK293T (human embryonic kidney 293 cells transformed with SV40 large T antigen, purchased from the American Type Culture Collection) cells transfected with pNL4-3 plasmid were employed to infect SupT1 (a human CD4 T cell line derived from NIH AIDS Reagent Program) using the spinoculation method (37). As shown in Figure 4C, HIV-RNA and p24 antigen were detected by RT-PCR and western blot in HIV-infected SupT1 cells in a dose-dependent manner, but not in uninfected cells. Importantly, compared to the uninfected control (lane 1), HIV-infected cells (lane 2 and 3) exhibited a reduced TRF2 expression at day 3 and day 6 after HIV-1 infection. In conjunction with telomeric DDR and cell apoptosis, these results suggest that TRF2 protein is inhibited in CD4 T cells by viral infection that is associated with the DDR-mediated cell apoptosis.

### KML001 Inhibits Shelterin TRF2 via the p53 and Sirt6-Mediated, Ubiquitin-Dependent Proteolysis

The mechanisms underlying TRF2 posttranscriptional inhibition remain unclear. We have recently shown that ubiquitin-mediated proteasome-degradation plays a key role in TRF2 regulation in CD4 T cells during HCV infection (38). In fibroblasts, the stability of TRF2 protein is also regulated via a p53-induced E3 ubiquitin ligase (46). Notably, p53 itself is regulated by proteasomal degradation in muscle cells (47). We thus hypothesized that this mechanism may also occur in KML001-treated T lymphocytes as a means of regulating TRF2 levels. To investigate the potential involvement of p53-dependent proteolysis in TRF2 degradation, we treated human CD4 T cells with 5 μM KML001 or DPBS for 48 h, followed by western blotting for p53 levels. As shown in Figure 4D, p53 protein levels were significantly upregulated by the KML001 treatment, indicating a strong DDR.

Additionally, a recent study indicates that the deacetylase Sirt6 specifically interacts with TRF2 and promotes its degradation via the ubiquitin-dependent proteolysis in response to DNA damage in fibroblasts (48). We thus examined Sirt6 expression and found a significant upregulation of its protein levels in KML001-treated CD4 T cells (Figure 4E); Also, Sirt6 upregulation was demonstrated in TCR-activated CD4 T cells exposed to the KML001 treatment (Figure 4F), suggesting that acetylation of TRF2 may also play a role in regulating TRF2 protein stability in T lymphocytes.

We further examined whether the protein degradation machinery would contribute to the observed TRF2 protein inhibition. To this end, we treated CD4 T cells with or without KML001 in the presence and absence of a proteasome inhibitor (MG132), which can prevent proteolysis process. Indeed, CD4 T cells exposed to KML001 exhibited an increase in p53 and Sirt6 expression, along with the decrease of TRF2 level (compare Figure 4G lane 1 and 2). While MG132 treatment further increased p53 and Sirt6 levels (Figure 4G lane 1 and 3), additional KML001 did not further increase their expressions (Figure 4G lane 2 and 4, likely due to test saturation), but the level of TRF2 appeared further decreased by the treatment. These results demonstrate that KML001 triggers telomeric DDR by suppressing shelterin TRF2 via promoting its protein degradation, likely through the p53 and Sirt6-mediated, ubiquitin-dependent proteolysis.

### KML001 Triggers Topological DDR in T Cells With Dynamic Activation and Depletion of ATM Kinase

**Figure 5:**
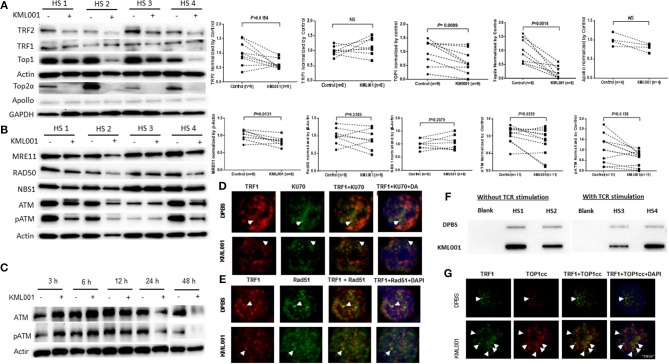
KML001 triggers topological DDR in CD4 T cells with dynamic activation and deficiency of ATM kinase. CD4 T cells were purified from HS and cultured in the presence or absence of KML001 for indicated time, followed by immunoblotting or confocal microscopic analysis. **(A)** Representative imaging and summary data of western blot analysis of TRF2, TRF1, and Top1 in CD4 T cells exposed to 5 μM KML001 or DPBS control for 48 h. Actin served as loading control. Since Top2a is only expressed in TCR-activated cells, Top2a and Apollo expressions were measured in CD4 T cells exposed to anti-CD3/CD28 stimulation (1 μg/ml) and 5 μM KML001 or DPBS treatment for 48 h. GAPDH served as loading control. **(B)** Representative imaging and summary data of western blot analysis of MRE11, RAD50, NBS1, ATM, and pATM expressions in CD4 T cells exposed to 5 μM KML001 or DPBS control for 48 h. **(C)** Western blot analysis of dynamic alterations in ATM and pATM expressions in CD4 T cells treated with 5 μM KML001 or DPBS control for 3, 6, 12, 24, and 48 h. **(D,E)** Representative imaging of confocal microscopic examination of co-localization of Ku70 and TRF1, as well as RAD51 and TRF1 in CD4 T cells, treated with KML001 or DPBS for 48 h. **(F)** TOP1cc detected in genomic DNA isolated from CD4 T cells treated with or without TCR stimulation and KML001 treatment. **(G)** Representative imaging of confocal microscopic examination of co-localization of TOP1cc and TRF1 in CD4 T cells treated with KML001 or DPBS control.

How KML001 triggers telomeric DNA damage that remains unrepaired has been rather unclear. The intertwined, helical structure of two complementary DNA strands is a unique feature of chromosomes that can cause DNA entanglements during genetic transactions, but topoisomerases can resolve these topological problems to ensure normal genetic activity and cell function (49). While catalytic inhibition of topoisomerase has been exploited to kill bacterial or cancer cells (50), the role of topoisomerases in reprogramming telomeric DDR and remodeling T cell functions remains largely unknown. Recently, we have discovered that the expressions and activities of Top1 as well as Top2a were significantly inhibited in CD4 T cells derived from chronically HCV, HBV, or HIV-infected individuals, leading to topological DNA damage, cell apoptosis, and ultimately, alterations in T cell homeostasis (unpublished data). Also, it has been shown that TRF2 can protect telomeres against topological DNA damage by recruiting Top2a and its binding partner Apollo (a member of the metallo-β-lactamase family that is required for telomere integrity in S phase) during replicative stress (22). To further investigate the mechanisms involved in KML001-induced DNA damage, we examined Top1 as well as Top2a levels (only expressed in activated T cells) in CD4 T cells exposed to KML001 for 48 h without or with anti-CD3/CD28 stimulation. As shown in Figure 5A, along with the inhibition of TRF2 (but not TRF1), the levels of Top1 and Top2a (but not Apollo) were significantly inhibited. These findings suggest that Top1/2a-mediated topological problems may involve in modulating T cell telomeric DNA damage.

To gain insight into the cause and consequence of telomeric DDR, we further explored the DNA repair machineries in CD4 T cells exposed to KML001. A major sensor of DNA-DSB is the MRN complex (MRE11, RAD50, and NBS1), which subsequently recruits the protein kinase ATM, an enzyme critically involved in regulating DNA repair for cell survival. We thus examined the ATM signaling pathway in KML-treated T cells. As shown in Figure 5B, DNA-DSB sensor MRE11, but not RAD50 or NBS1, was significantly inhibited in CD4 T cells exposed to KML001. In addition, ATM expression and phosphorylation (pATM) were markedly suppressed in T cells with the KML001 treatment for 48 h.

Since accumulation of DNA damage usually activates the protein kinase ATM, which is in contrast with our findings of ATM deficiency, we examined the kinetics of ATM expression and activation at different time-points following the KML001 treatment. As shown in Figure 5C, ATM and pATM were upregulated in the early phase (3~6 h), and then downregulated in the late phase (24~48 h), after KML001 treatment, reflecting a dynamic alteration (early activation followed by depletion) of this DNA repair kinase in the process of DDR in T cells. These results are in line with our findings that insufficiency of ATM and TRF2 promotes CD4 T cell loss in chronic HCV infection (36, 38) and in latent HIV infection (unpublished data).

### KML001 Induces TOP1cc Accumulation to Prevent DNA Repair Molecule Recruitment to the DNA Damage Sites

We have recently shown that the NHEJ pathway, rather than the homologous recombination (HR) pathway of DNA repair, participates in the ATM-mediated telomeric DNA damage repair (unpublished data). However, how DSBs accumulate in cells despite the presence of DNA repair proteins remains unknown. It is well-known that Ku70 and Ku80 make up the Ku heterodimer, which bind to DSB ends and is required for the NHEJ pathway of DNA repair. It is also required for V(D)J recombination, which utilizes the NHEJ pathway to promote antigen/antibody diversity in the mammalian immune system. In addition to its role in the NHEJ-mediated DNA repair, Ku is involved in telomere maintenance (51). It has been reported that Ku70 deficient mice exhibit premature aging (52), suggesting that Ku70 plays an important role in longevity assurance and that reduced ability to repair DNA DSB or maintain telomere integrity causes early aging. To determine the role of the NHEJ pathway in repairing KML001-induced telomeric DNA damage, we measured the Ku70-dependent, dysfunctional TIF in CD4 T cells exposed to KML001 or DPBS for 48 h by confocal microscopy. Surprisingly, we could not identify any Ku70/TRF1 co-localization in cells treated with KML001 or DPBS control (Figure 5D), suggesting that Ku70 failed to be recruited to the damaged telomeres. We also examined possible involvement of the HR pathway in KML001-induced DSB repair. Since RAD51 is involved in the search for homology and strand pairing stages of the process (53, 54), we used confocal microscopy analysis for the RAD51/TRF1 co-localization in CD4 T cells exposed to KML001 or DPBS for 48 h, and again, we did not find any difference between the two treatments (Figure 5E).

Topoisomerases cleave and rapidly reseal one (type I) or two (type II) DNA strands, generating a transient break through which topological modifications can occur (49). This catalytic process is rather “dangerous” in that it generates an intermediate in which the topoisomerase becomes covalently linked to the terminus of nucleic acids through a phosphotyrosine linkage, designated as the topoisomerase cleavage complex (TOPcc). Failure to complete this catalytic cycle results in trapping of topoisomerase on DNA termini, generating protein-linked DNA breaks (PDB)—a frequent event that causes cell death (55, 56). To determine if KML001-induced telomeric DNA damage is due to TOPcc trapped in T cell chromosomes, we measured TOP1cc in genomic DNA of CD4 T cells exposed to KML001 or DPBS control, using a monoclonal antibody that specifically recognizes covalent TOP1-DNA complexes, but not free TOP1 or DNA, by immunoblotting (57). As shown in Figure 5F, a significant amount of TOP1cc was detected in CD4 T cells exposed to KML001 for 48 h with or without TCR stimulation. These results suggest that KML001-induced dysfunctional, senescent T cells have topological aberrancies, i.e., TOP1 inhibition, leading to TOP1cc accumulation.

Since KML001 induces cytotoxicity by trapping TOP1cc at the TOP1-DNA covalent sites, which in turn interacts with advancing replication or transcription complexes to generate lethal DNA lesions, we further hypothesized that TOP1cc may accumulate and occupy the DSB ends, preventing DNA repair molecules to be recruited to the damaged sites of T cells. To test this possibility, we examined the KML001-induced, TOP1cc-mediated dysfunctional TIF using a monoclonal antibody that specifically recognizes covalent TOP1-DNA complexes, but not free TOP1 or DNA, by confocal microscopy (57). Indeed, we found a significant increase of TOP1cc/TRF1-formed TIFs in T cells exposed to KML001 compared to the DPBS control (Figure 5G). Additionally, we found TOP1cc accumulation at damaged telomeres in T cells treated with CPT (a specific TOP 1 inhibitor that can trigger topological DNA damage by inducing TOP1cc accumulation at genomic DNA), whereas not in ATM inhibitor (KU60019)-induced DNA damage, which does not involve TOP1cc induction but occurs via the NHEJ pathway for DNA damage repair (unpublished data). Taken together, these results suggest that KML001 induces TOP1cc accumulation and trap at the DSB sites that may prevent DNA repair molecule recruitment and inhibit DNA damage repair.

### T Cells From Virus-Infected Individuals Are More Vulnerable to Telomere Loss-Induced Apoptotic Death

**Figure 6:**
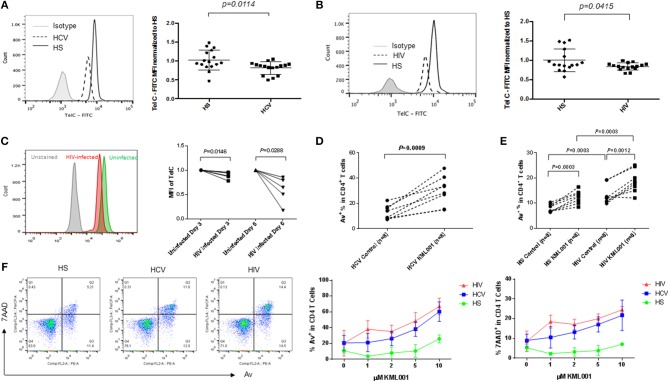
CD4 T cells derived from HCV or HIV-infected individuals are more vulnerable to telomere loss-mediated apoptosis. **(A,B)** Telomere length, measured was by Flow-FISH, in CD4 T cells isolated from HCV- or HIV-infected individuals and HS. Representative overlaid histograms and summary data with mean ± SEM and *p*-values are shown. **(C)** Representative overlaid histogram and summary data of Flow-FISH analysis for telomere length in SupT1 cells infected with or without HIV-1 for 3 and 6 days. The mean fluorescence intensity (MFI) of telomere length in HIV-infected cells are normalized by those in uninfected cells. **(D)** PBMCs derived from chronically HCV-infected individuals were treated with 5 μM KML001 or DPBS control for 24 h, followed by flow cytometry analysis of Av expression in CD4^+^ T cells. **(E)** PBMCs derived from latently HIV-infected individuals and HS were treated with 5 μM KML001 or DPBS control for 24 h, followed by flow cytometry analysis of Av expression in CD4^+^ T cells. Summary data with mean ± SEM (*n* = 8) and *p*-values are shown. **(F)** Dose-dependent induction of apoptotic death of CD4 T cells isolated from HCV- and HIV-infected individual and HS (*n* = 3 in each group) and exposed to varying concentrations of KML001 for 12 h. Representative dot plots of CD4^+^ T cells at 2 μM KML001 treatment and summary data of the dynamic changes of Av/7AAD levels in CD4^+^ T cells following varying concentrations of KML001 treatment are shown.

We have previously demonstrated that chronically HCV and HIV-infected individuals exhibit T cell exhaustion and senescence (1–4). Since the hallmark of T cell senescence is telomere shortening, we measured telomere length in CD4 T cells isolated from HCV and HIV-infected individuals using Flow-FISH. As shown in Figures 6A,B, similar to the CD4 T cells treated by KML001 (Figure 3D), telomere attrition was detected in CD4 T cells derived from both HCV and HIV-infected individuals. This is consistent to our findings with HCV samples reported previously (4, 38). We also measured the telomere length in SupT1 cells with or without HIV-1 infection *in vitro*. As shown in Figure 6C (representative overlaid histogram and dynamic Flow-FISH data), T cells infected with HIV-1 at 3 days, and particularly at 6 days, showed a clearly shortened telomere length compared to uninfected cells. These data recapitulate the findings with CD4 T cells from HIV-infected patients.

Since KML001 can bind to telomeric DNA sequences, causing telomere erosion and cell apoptosis (32), we also measured apoptosis of CD4 T cells derived from chronically HCV- and HIV-infected individuals with or without KML001 treatment. As shown in Figures 6D,E, HCV- and HIV-derived T cells were very sensitive to the KML001-induced cell apoptosis. We next sought to determine whether CD4 T cells derived from chronically virus-infected patients, which we have shown that they exhibit a senescent state with an inhibited TRF2 and shortened telomeres, are more sensitive to KML001-induced apoptosis. To this end, we treated CD4 T cells derived from virus-infected individuals and age-matched HS with varying concentrations (0, 1, 2, 5, 10 μM) of KML001 for a short period of time (12 h), followed by flow cytometric analysis for Av/7AAD expression. As shown in Figure 6F, compared to age-matched HS, HCV- and HIV-derived T cells exhibited a relatively higher percentage of apoptotic (Av^+^) and dead (7AAD^+^) cells following the KML001 treatment, in a dose-dependent manner, suggesting that they are more vulnerable to the KML001-induced cell apoptotic death.

Taken together, our data reported here suggest that CD4 T cells from chronically virus-infected individuals are prone to apoptosis and susceptible to telomere insult, likely due to the loss of telomere integrity caused by the impairment of the telomere sheltering (TRF2 protection), elongation (telomerase activity), topology (Top1/2a disentanglement), and DNA repair (ATM kinase) machineries.

## Discussion

During chronic viral infections, T cells exhibit a dynamic over-activation and proliferation due to persistent antigenic stimulation, followed by exhaustion and senescence, resulting in immune dysfunctions that contribute to persistent infection and vaccine non-responsiveness. Senescent T cells are characterized by accelerated telomere erosion, which has two potential outcomes: cell cycle arrest or cell apoptosis. Specifically, if telomere attrition is mild to moderate, it will trigger cell cycle arrest in order to give the cell a chance to repair the DNA damage; however, if the telomere loss reaches a critical point or DNA damage becomes irreparable, it will trigger programmed cell death to get rid of cells harboring unhealthy genetic transformations.

**Figure 7:**
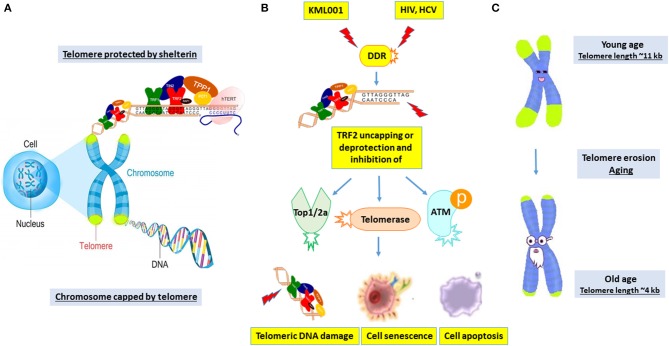
A working model depicting the novel machineries involved in disrupting telomere integrity to induce T cell senescence and apoptosis during chronic viral infection. **(A)** The telomere cap on the end of chromosomes unravels progressively with every cell division. The shelterin complex is required to protect telomeres against undesired DNA damage response (DDR). **(B)** KML001 and chronic viral (HCV or HIV) infection can induce shelterin protein TRF2 degradation and inhibition of Top2a, telomerase, and ATM enzyme activities, leading to telomeric DNA damage, T cell senescence and apoptosis. **(C)** Telomere shortening is a normal process in T cells with human aging. While many factors drive human aging, viral infection accelerates telomere erosion, resulting in premature T cell aging and immune senescence. This represents a novel molecular mechanism underlying the T cell dysfunction and immune (vaccine) non-responsiveness seen in patients with chronic viral infection.

The mechanisms of telomere erosion and its role in cell survival during genomic insult and cell stress remain unclear. This is particularly true for T lymphocytes that undergo self-renewal through proliferation upon antigenic challenge to fight against invading pathogens. Notably, human telomeres are programmed to lose 50~100 base pairs (bp) per population doubling (PD), resulting in replicative senescence after ~50 PDs (17). This shortening rate can reach ~250 bp during viral infection, suggesting accelerated nucleolytic attack or DNA damage on chromosome ends (17). Thus, we speculate that senescence occurs when T cell chromosomes experience significant telomere loss. However, telomere integrity is preserved and safeguarded by multiple machineries (14–18). Although the accidentally generated DNA-DSB can induce DDR, natural chromosome ends (telomeres) do not trigger this response. We now know that this suppression of DDR involves the telomere protecting proteins (shelterin) that can inhibit unwanted DDR (Figure 7A). Since the Nobel Prize-winning discovery of the enzyme telomerase in 1984, identifying other biological molecules that lengthen or shorten the protective caps on the ends of chromosomes has been slow going. While it is assumed that the relatively normal hTERT levels we found during viral infection could compensate for telomere loss to prevent cell crisis, the data presented in this study using CD4 T cells treated with KML001 or derived from chronic viral infection argue against this notion, and suggest that telomerase is not the only factor involved. Rather, the main events heralding the end of the replicative life of human T cells are a failure of several key players involved in protecting telomere integrity, i.e., TRF2 deprotection and its associated loss of telomerase, topoisomerase, and ATM kinase activities (Figure 7B). Since telomeres are critical to maintain chromosome integrity and keys to human health and longevity (15–17), the machineries involved in telomere erosion are therefore major potential anti-aging targets (Figure 7C).

TRF2 is a sequence-specific DNA binding protein that binds to the duplex array of TTAGGG repeats at human telomeres and protects chromosome ends from end-to-end fusion (19, 20). Loss of TRF2 function leads to chromosomal abnormalities, cell cycle arrest, and activation of the ATM–p53-mediated DDR pathway (19, 20). The protective activity of TRF2 may be related to its ability to form t loops, in which the G strand invades the duplex part of the telomeres (19, 20). Based on our studies showing that telomeres are not only shortened, but DNA is also damaged, we believe that premature T cell aging is induced by a change in the protected status (integrity) of telomeres rather than by a pure loss of telomeric DNA. This hypothesis led us to explore how TRF2 deprotection affects telomere integrity and cellular fate in chronic viral infection. Remarkably, our results derived from KML001-treated CD4 T cells are similar to the findings in T cells during chronic viral (HCV, HIV) infection in that these CD4 T cells show a senescent status, characterized by telomere erosion due to TRF2 inhibition via the p53- and Sirt6-mediated ubiquitin degradation, and are more vulnerable to telomere loss-mediated cell apoptosis. This is consistent with our recent findings in chronic HCV infection, in which the expressions of p53 and E3 ubiquitin ligase Siah-1a were significantly increased in CD4 T cells upon proteasome inhibition by MG132 treatment. In addition, p21^cip1^, a p53 downstream cell cycle inhibitor and cell senescence marker, was significantly upregulated by MG132 treatment in HCV-derived T cells, suggesting a p53-dependent, Siah1a-mediated ubiquitin degradation of TRF2 in senescent T cells during viral infection (38). Importantly, silencing TRF2 in healthy T cells by transfecting its siRNA results in telomeric DNA damage. Conversely, reconstituting TRF2 in T cells from virally infected individuals leads to a decrease in γH2AX level, whereas overexpressing a truncated TRF2 mutant results in an increase of γH2AX (38). Taken together, these findings suggest that TRF2 sheltering is essential for protecting the telomere from DNA damage and securing T cell survival. Our results in which we show normal expression levels of TRF1 and telomerase expression in CD4 T cells exposed to KML001 and HCV or HIV infection confirm the idea that TRF1 controls telomere dynamics primarily by affecting the telomerase-mediated telomere elongation (58, 59). Thus, TRF1 and TRF2 both act as negative regulators of telomere length, but affect different aspects of telomere dynamics.

The role of telomere uncapping in cell senescence is incompletely understood. A recent study indicates that replicative senescence is induced by partially deprotected telomeres, which activate an ATM and topoisomerase-mediated DDR without chromosomal end-to-end fusions (60). This telomere deprotection response is functionally distinct from the genomic DDR in that, unlike genomic DNA breaks, deprotected telomeres do not contribute to the G2/M checkpoint and are instead passed through cell division to induce p53-dependent G1 arrest in daughter cells (60). Telomere deprotection is therefore an epigenetic signal passed between cell generations to ensure that replication-dependent growth arrest occurs in stable diploid G1 phase cells, before genomic instability can occur. Moreover, TRF2 can reduce the senescence set-point, defined as telomere length at senescence ranging from 7 to 4 kilobases (61). TRF2 can protect critically short telomeres and repress chromosome-end fusion, which explains the ability of TRF2 to delay senescence. If genomic insult is persistent and critical, the cells will be in crisis, requiring a bypass of senescence pathways through loss of checkpoints and causing TRF2 uncapping and further telomere shortening and spontaneous telomere fusion, resulting in programmed cell death (apoptosis). On the other hand, retroviral-mediated overexpression of TRF2 in several human fibroblast cell lines results in an accelerated telomere shortening, likely through an enhanced 5′ exonucleolytic processing of telomere ends by recruiting an exonuclease regardless of the status of p53 or the p16-RB pathways; the cells remain alive, however, without accelerating senescence (61). These studies suggest that excess TRF2 can protect very short telomeres to maintain cell viability and that this is the mechanism by which TRF2 can extend the lifespan and alter the senescence set-point of primary human cells.

Collectively, our findings support that replicative senescence in human T cells is caused by a change in the status of the telomeric complex, in particular TRF2 loss-mediated telomeric DNA damage and failure to repair, rather than by a purely telomerase-mediated loss of telomeric sequences. If the senescence signal was a function of cell chromosome ends lacking telomeric DNA altogether, TRF2 would not be expected to repress this signal and alter the senescence set-point. One possibility is that critically shortened telomeres in senescent human cells can no longer bind sufficient TRF2 to achieve a protective state, such as the t-loop. Alternatively, binding of TRF2 may facilitate the recruitment of other proteins required for telomere protection and suppression of senescence. For instance, TPP1 reduction has been shown to cause telomere attrition and cellular senescence via Sirt1 deacetylase-mediated proteolysis degradation (62). Intriguingly, we have recently reported that the anti-aging molecule Sirt1 is upregulated in CD4 T cells via a decline in miRNA181a expression to balance DUSP6-mediated cell senescence during chronic HCV infection (3, 4). We find that while the mRNA level of TPP1 is upregulated, its protein expression in CD4 T cells is downregulated by KML001 treatment, as well as by HCV infection (38). Notably, TPP1 can directly interact with telomerase, whereas TRF2 can bind TIN2 and then TPP1 to form a sub-complex (38). Whether the miRNA181a/Sirt1 axis plays a role in TPP1 posttranscriptional regulation and whether TRF2/TIN2/TPP1 fails to recruit telomerase at telomeres during viral infection is under investigation in our laboratory.

This study has clinical relevance and potential applications: first, our data showed that T cell apoptotic death during chronic viral infection correlated with TRF2-mediated telomere uncapping and its associated telomerase, topoisomerase, and ATM kinase inhibition. Based on this, we propose that TRF2 inhibition is the main mechanism that limits cellular lifespan upon bypass of senescence. Aging is a progressive mystery involving a range of architects, but TRF2-mediated telomere shortening has been associated with multiple age-related diseases and thus we believe that we have to find ways to replenish TRF2 and repair telomeres if we want to live healthy and longer. Second, these findings might also offer a translational opportunity for tumorigenesis, anti-cancer treatment, and immunomodulation, since exacerbation of TRF2-mediated telomere deprotection sensitizes cancer and immune cells to telomere-targeting drugs, such as arsenic compounds. Additionally, uncovering the telomere mystery can help us understand how cells strike a balance between premature aging and the uncontrolled cell growth of cancer, which is very intriguing. Third, cell cycle arrest has been associated with chronic infections in checkpoint-compromised cells (63). Similarly, bone marrow failure and neurodegeneration in individuals with telomere loss are frequent, which could potentially be explained by cell progression arrest that results from relatively unprotected telomeres (64, 65). Therefore, TRF2-mediated telomere uncapping and the resulting telomere loss-driven cell cycle arrest in the early stage of viral infections may function as a double-edged sword, explaining both the overwhelming cell death storm in acute infection, and the immune tolerance or immunosuppression in chronic/persistent infection; whereas lengthened and damaged telomeres are associated with cell chromosomal rearrangement and tumors (66). Thus, this study enhances our understanding of T cell senescence, apoptosis, and functional dysregulation during viral infection, and identifies novel targets to modify telomeric shelterin, particularly TRF2-mediated telomeric DNA damage and repair machineries, as a new strategy to prevent chromosomal ends from unwanted DDR and to salvage cells from crisis.

## Data Availability

All datasets generated for this study are included in the manuscript and/or the supplementary files.

## Ethics Statement

The study protocol was approved by the institutional review board (IRB) of East Tennessee State University and James H. Quillen VA Medical Center (ETSU/VA IRB, Johnson City, TN). Written informed consent was obtained from all participants.

## Author Contributions

DC performed most of the experiments. JZ, LNTN, LNN, SK, XD, MS, and BT participated in some experiments. XW and ZM provided technical support. YZ, ME, SN, LW, and JM offered intellectual input for troubleshooting and discussion of the findings. ZY supervised the project and wrote the manuscript, with the help of all other authors.

### Conflict of Interest Statement

The authors declare that the research was conducted in the absence of any commercial or financial relationships that could be construed as a potential conflict of interest.
